# Artificial-intelligence-based computed tomography histogram analysis predicting tumor invasiveness of lung adenocarcinomas manifesting as radiological part-solid nodules

**DOI:** 10.3389/fonc.2023.1096453

**Published:** 2023-02-23

**Authors:** Jian Gao, Qingyi Qi, Hao Li, Zhenfan Wang, Zewen Sun, Sida Cheng, Jie Yu, Yaqi Zeng, Nan Hong, Dawei Wang, Huiyang Wang, Feng Yang, Xiao Li, Yun Li

**Affiliations:** ^1^ Department of Thoracic Surgery, Peking University People’s Hospital, Beijing, China; ^2^ Thoracic Oncology Institute, Peking University People’s Hospital, Beijing, China; ^3^ Department of Radiology, Peking University People’s Hospital, Beijing, China; ^4^ Department of Thoracic Surgery, Qingdao Women and Children’s Hospital, Qingdao, China; ^5^ Institute of Advanced Research, Infervision Medical Technology Co., Ltd, Beijing, China

**Keywords:** lung adenocarcinoma, CT histogram, part-solid nodule, tumor invasiveness, three-dimensional index

## Abstract

**Background:**

Tumor invasiveness plays a key role in determining surgical strategy and patient prognosis in clinical practice. The study aimed to explore artificial-intelligence-based computed tomography (CT) histogram indicators significantly related to the invasion status of lung adenocarcinoma appearing as part-solid nodules (PSNs), and to construct radiomics models for prediction of tumor invasiveness.

**Methods:**

We identified surgically resected lung adenocarcinomas manifesting as PSNs in Peking University People’s Hospital from January 2014 to October 2019. Tumors were categorized as adenocarcinoma *in situ* (AIS), minimally invasive adenocarcinoma (MIA), and invasive adenocarcinoma (IAC) by comprehensive pathological assessment. The whole cohort was randomly assigned into a training (70%, n=832) and a validation cohort (30%, n=356) to establish and validate the prediction model. An artificial-intelligence-based algorithm (InferRead CT Lung) was applied to extract CT histogram parameters for each pulmonary nodule. For feature selection, multivariate regression models were built to identify factors associated with tumor invasiveness. Logistic regression classifier was used for radiomics model building. The predictive performance of the model was then evaluated by ROC and calibration curves.

**Results:**

In total, 299 AIS/MIAs and 889 IACs were included. In the training cohort, multivariate logistic regression analysis demonstrated that age [odds ratio (OR), 1.020; 95% CI, 1.004–1.037; *p*=0.017], smoking history (OR, 1.846; 95% CI, 1.058–3.221; *p*=0.031), solid mean density (OR, 1.014; 95% CI, 1.004–1.024; *p*=0.008], solid volume (OR, 5.858; 95% CI, 1.259–27.247; *p* = 0.037), pleural retraction sign (OR, 3.179; 95% CI, 1.057–9.559; *p* = 0.039), variance (OR, 0.570; 95% CI, 0.399–0.813; *p*=0.002), and entropy (OR, 4.606; 95% CI, 2.750–7.717; *p*<0.001) were independent predictors for IAC. The areas under the curve (AUCs) in the training and validation cohorts indicated a better discriminative ability of the histogram model (AUC=0.892) compared with the clinical model (AUC=0.852) and integrated model (AUC=0.886).

**Conclusion:**

We developed an AI-based histogram model, which could reliably predict tumor invasiveness in lung adenocarcinoma manifesting as PSNs. This finding would provide promising value in guiding the precision management of PSNs in the daily practice.

## Introduction

As the low-dose chest computed tomography (CT) is becoming more popular for lung cancer screening, subsolid nodules (SSNs) are increasingly being detected as an important part of the clinical work of thoracic surgeons (1, 2). SSNs are classified into pure GGN (pGGN) and part-solid nodule (PSN) depending on the presence of solid components (3). Pathologically, adenocarcinoma *in situ* (AIS), minimally invasive adenocarcinoma (MIA), and invasive adenocarcinoma (IAC) can appear on CT images as persistent PSNs (4). The 5-year recurrent-free survival (RFS) after complete resection of AIS and MIA is close to 100%, while the 5-year RFS of stage I IAC is only 74.1% (5). In terms of the extent of surgical resection, sublobar resection, including pulmonary wedge resection and segmentectomy, is mainly recommended for AIS/MIA (6), which can preserve the pulmonary parenchyma and effectively reduce surgery-related complications. Lobectomy and lymph node dissection are required for IAC to pursue a lower tumor recurrence rate with a higher rate of surgical complications. Therefore, the accurate preoperative imaging evaluation of PSN to predict IAC is important to guide clinical treatment decisions, especially for the extent of surgical resection.

In recent years, radiomics had made remarkable progress in identifying the degree of invasiveness in pulmonary lesions suspicious of lung cancer (7, 8). Radiomic parameters only or combined radiomics parameters and clinical features had been utilized to construct prediction models of IAC among SSNs with good diagnostic performance (area under curves, 0.72–0.98) (9–13). Only three studies focused on the assessment of invasiveness among PSN group with a relatively small sample (9, 12, 13). Moreover, most of the above studies used manually time-consuming segmentation, which was difficult to be applied in a real-world setting.

As a convenient and representative method of radiomics, the voxel-based CT histogram could provide some key radiological factors beyond human eyes such as skewness and entropy (7, 14, 15). Recently, voxel-based histogram analysis of chest CT has shown great usefulness in identifying pathological invasiveness, lymph node status prediction, and early-stage lung adenocarcinoma suitable for sublobar resection (16–18). However, several above studies included all radiological types of early-stage lung adenocarcinoma not only SSNs, and several studies about SSNs were limited by a small sample. To our knowledge, no published studies had focused on the part-solid nodule, which needs surgical treatment among SSN subtypes. Furthermore, as a high-efficiency and promising automatic method, artificial intelligence (AI) technology has not been integrated in this diagnostic field to date.

In this study, we proposed to use CT histograms based on novel deep-learning artificial intelligence technology to explore clinical and radiomic indicators that effectively distinguish IAC from AIS/MIA in PSNs with a large sample and to construct a prediction model for the invasiveness of PSNs, which can help guide clinical treatment decisions.

## Methods

### Study population and selection criteria

**Figure 1:**
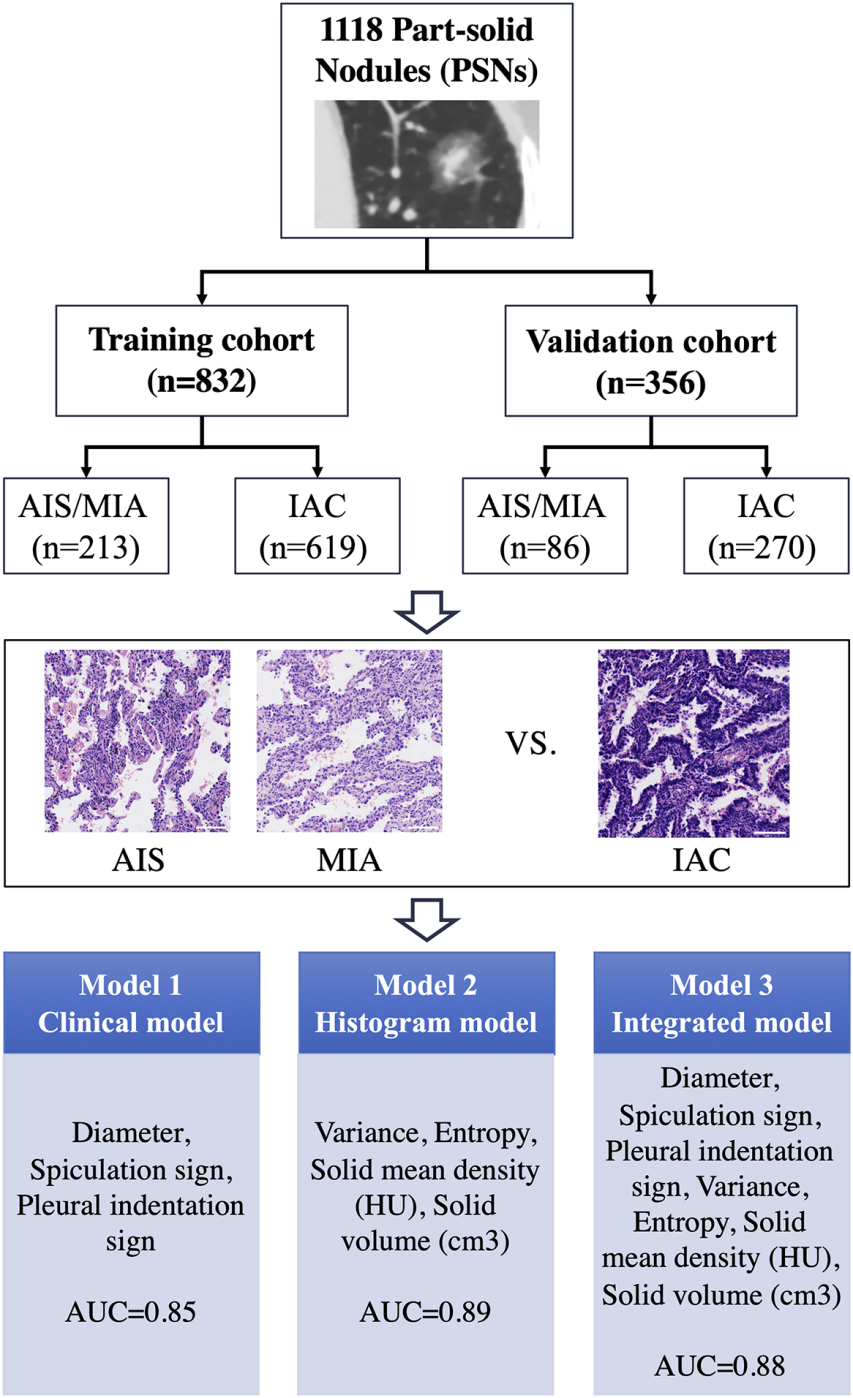
Overall study design process for the training and validation cohorts and prediction performance by each model for predicting invasive adenocarcinoma (IAC). AIS, adenocarcinoma *in situ*; MIA, minimally invasive adenocarcinoma; AUC, area under the ROC curve. The length of the ruler in the figure is 10 nm.

This study was a cross-sectional retrospective study. Consecutive patients with chest CT suggestive of PSNs (total diameter, 5–30 mm) and received surgeries at the Department of Thoracic Surgery, Peking University People’s Hospital from January 2014 to October 2019 were included in this study. The determination of PSNs was performed by two experienced radiologists (Qingyi Qi and Yaqi Zeng). Postoperative pathological findings confirmed 1,188 lung nodules as primary lung adenocarcinoma (**Figure 1**). The corresponding clinical information, pathological information, and imaging information were collected through the electronic medical record system.

### CT image acquisition

All patients’ chest CT images were retrospectively collected with the same imaging acquisition parameters: scans with the collimation of 64×0.625 mm, tube voltage of 120 kVp, tube current modulation, gantry rotation speed of 0.5 s/r, and 1.0/1.25 mm reconstructed slice thickness with lung window setting (HU) of (1,600, −600) and mediastinal window (HU) setting of (400, 40) using a 256-row CT scanner (Revolution CT, GE Healthcare, America). All thin-layer images were transmitted to the Lung Nodule Artificial Intelligence Intelligent Assisted Diagnosis System (InferRead CT Lung, Infervision Medical Technology Co., Ltd.) for automatic detection of all lung nodules. The nodules screened by the InferRead system were individually verified by two experienced radiologists, and the PSNs that needed to be surgically resected were manually screened.

### Evaluation of voxel-based histogram features

**Figure 2:**
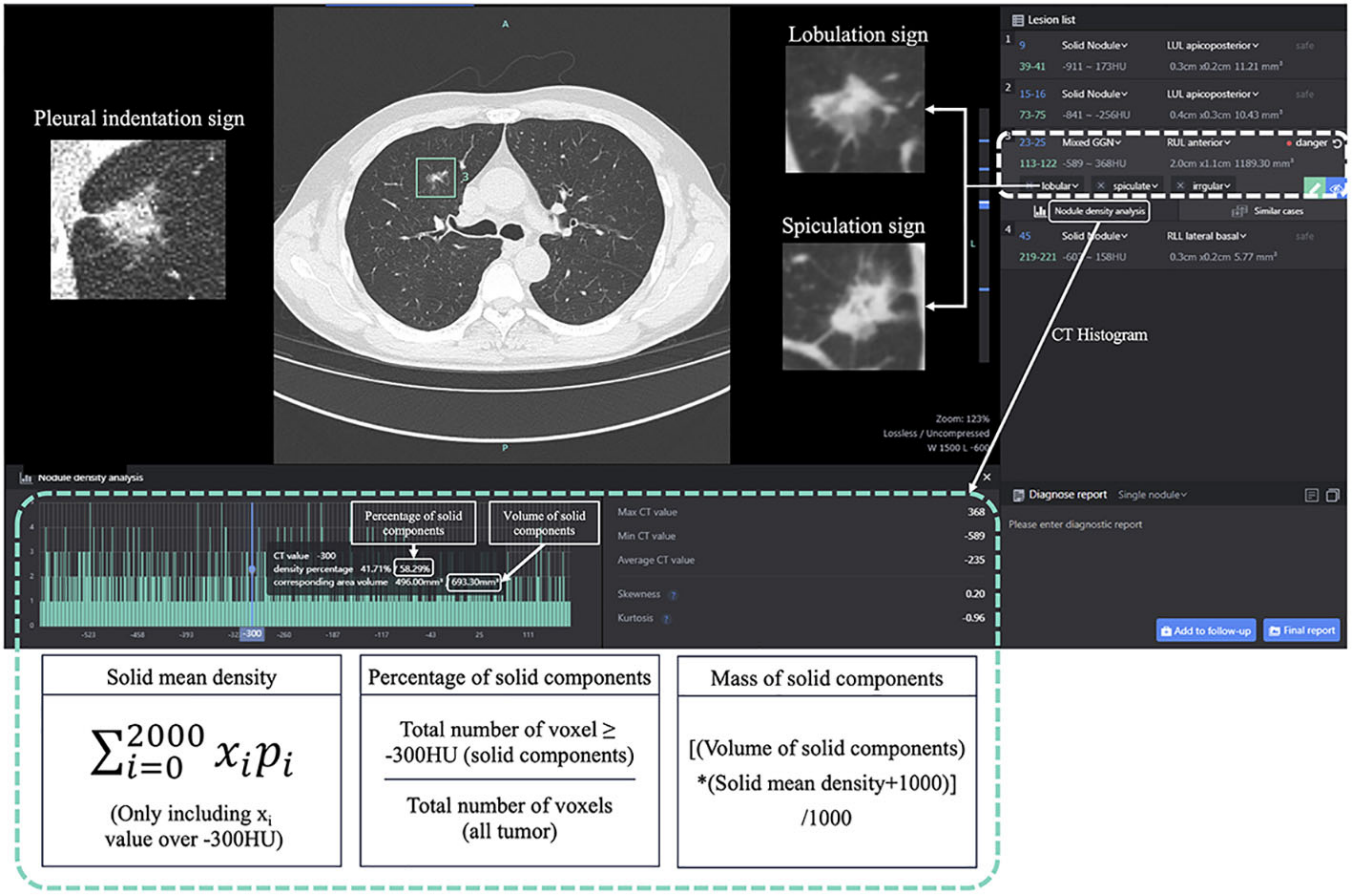
The CT histogram parameters using InferRead CT Lung, an AI-based pulmonary nodule auxiliary diagnosis system. Mixed GGN, mixed ground-glass nodule.

The lung nodule artificial-intelligence-assisted diagnosis system is based on a deep learning algorithm to achieve automatic segmentation of the range of ground glass nodules and recognition of typical signs (19–21). In the whole calculation process, the system automatically divides the range of the ground glass nodules and calculates the number of voxels corresponding to each CT value in the whole SSN. Each CT value and the corresponding number of voxels are stored as a LIST, and the LIST of the whole nodule is stored as a DICTIONARY. The information obtained is used to calculate the required index by the corresponding formula. First, the CT value threshold of −300 HU was used to distinguish the solid component from the ground glass component. The nodule volume, mean density, solid component volume, percentage of solid component, mass, mass of solid components, and other three-dimensional metrics were calculated based on the voxel method and the corresponding formulas, as follows (**Figure 2**).

Solid mean density = $\sum_{i=0}^{2000}x_{i}p_{i}$ (Only including x_i_ ≥ -300HU).

Percentage of solid components= total number of voxel ≥ −300 HU (solid components)/total number of voxels (all tumor)

Mass= [nodule volume×(mean density +1,000)]/1,000.

Mass of solid components= [solid components volume× (solid mean density +1,000)]/1,000.

Then, the CT histograms were constructed based on the number of voxels corresponding to each CT value in the nodule range. Variance, skewness, kurtosis, entropy, and other density histogram-related indicators were automatically calculated by python coding and the corresponding formulae. Meanwhile, the typical signs detected and identified by the system were confirmed by two radiologists as morphological indicators, including lobar signs, spiculation signs, and pleural traction signs.

### Construction of the prediction model

The whole cohort was randomly assigned into a training (70%, n=832) and a validation cohort (30%, n=356) to establish and validate the prediction model. This ratio (7:3) ensured the maximal utilization of the data for constructing a predictive model with a considerable number of sample size for validation (**Figure 1**). Logistical regression analysis based on the training cohort was performed to evaluate the odds ratio (OR) and to assess the parameters’ ability to predicting the risk of IAC. Variables with *p*-value <0.1 selected in the univariate analysis were included into the multivariate analysis. The variables with significant clinical meanings and the parameters with *p*-value <0.05 in the multivariate logistical regression analysis were used to establish three predictive models: clinical model (Model 1), histogram model (Model 2), and integrated model (Model 3).

The predictive models were subjected to a 10-fold cross-internal validation within the training cohort and independent validation in the validation cohort. Calibration plots were drawn to evaluate the goodness of fit of models, and the discriminative ability was assessed by receiver operating characteristic (ROC), the area under the curve (AUC), Akaike information criterion (AIC), and Bayesian information criterion (BIC). Higher AUC and lower AIC/BIC values represent higher discriminative ability. Delong’s test was performed to compare the AUCs of different models.

### Statistical analysis

Statistical analyses were performed using SPSS 26.0 software and R software version 3.6.0. Categorical variables were analyzed by Pearson chi-square test, continuous variables that conformed to a normal distribution were analyzed by independent samples t-test, and the part that did not conform to a normal distribution was analyzed by rank sum test (Mann–Whitney U test). Variables with *p*<0.1 in the univariate analysis were included in the multi-factor logistic regression analysis, and the assessment model of IAC in PSN was constructed. The “caret” package in R software was used to depict the calibration plots, and the “pROC” package was utilized to draw the ROC curve, to calculate the AUC, sensitivity, and specificity of the ROC curve, and to perform the Delong’s test. The “stats” package was used to calculate the AIC and BIC values. All statistical tests were two-tailed, and a *p*-value <0.05 was considered statistically significant.

## Results

### Clinical characteristics and histopathological nodule features

Among all included PSNs, 30 cases (2.5%) were AIS, 269 cases (22.6%) were MIA, and 889 cases (74.8%) were IAC (**Figure 1**). Women were the majority (66.5%) of all PSNs. Only a relatively small number of patients had a combined history of smoking (18.5%), previous malignancy (6.8%), and family history of malignancy (12.4%). The mean age of IAC group (60.31) was significantly greater than that of the AIS/MIA group (55.74), and the proportion of smokers was significantly higher than that of the AIS/MIA group. There was no statistical difference between the two groups in terms of previous history of malignancy and family history of malignancy (**Table 1**).

**Table 1:** Comparison of clinical information of AIS/MIA and IAC in 1,188 cases of PSNs.

Features	AIS/MIA	IAC	*p*-value
Gender			0.085
Women	211 (70.6)	579 (65.1)	
Male	88 (29.4)	310 (34.9)	
Age (years)	55.74 ± 10.21	60.31 ± 9.94	<0.001
Smoking history			0.001
Non-smokers	263 (88.0)	705 (79.3)	
Current/previous smokers	36 (12.0)	184 (20.7)	
History of malignancy			0.369
Yes	17 (5.7)	64 (7.2)	
None	282 (94.3)	825 (92.8)	
Family history of malignant tumors			0.068
Yes	28 (9.4)	119 (13.4)	
None	271 (90.6)	770 (86.6)	

Data are expressed as mean ± standard deviation or as a number (percentage).

### Imaging features of the different pathological types in PSNs

In assessing the imaging metrics of PSN, we incorporated three-dimensional metrics such as density, volume, mass, and percentage of solid components; morphological metrics such as fractional lobe sign, spiculation sign, and pleural traction sign; and density histogram-related metrics such as variance, kurtosis, skewness, and entropy. The imaging information of the two groups is detailed in **Table 2**.

**Table 2:** Comparison of imaging features of AIS/MIA and IAC in 1,188 cases of PSNs.

Features	AIS/MIA	IAC	*p*-value
Average density (HU)	-483.07 ± 96.11	-408.40 ± 130.08	<0.001
Solid mean density (HU)	-212.83 ± 55.16	-168.19 ± 54.71	<0.001
Volume (cm^3^)	0.57 ± 0.68	1.96 ± 1.67	<0.001
Volume of solid components (cm^3^)	0.08 ± 0.17	0.59 ± 0.83	<0.001
Mass (g)	0.29 ± 0.35	1.17 ± 1.08	<0.001
Mass of solid components (g)	0.06 ± 0.15	0.51 ± 0.75	<0.001
lobar sign	105 (35.1)	685 (77.1)	<0.001
Spiculation sign	82 (27.4)	645 (72.6)	<0.001
Pleural traction sign	4 (1.3)	215 (24.2)	<0.001
Percentage of solid components (%)	14.00 ± 14.55	28.09 ± 22.55	<0.001
Density Histogram			
Variance (×10000)	1.87 ± 1.20	2.51 ± 1.23	< 0.001
Skewness	0.83 ± 0.49	0.69 ± 0.54	< 0.001
Kurtosis	3.39 ± 1.50	3.09 ± 1.49	0.003
Entropy	7.94 ± 0.55	8.63 ± 0.46	< 0.001

Data are expressed as mean ± standard deviation or as a number (percentage).

All imaging features were statistically different between the two groups. Mean density (−408.40 ± 130.08 vs. −483.07 ± 96.11, e<0.001), mean density of the solid component (−168.19 ± 54.71 vs. −212.83 ± 55.16, e<0.001), volume (1.96 ± 1.67 vs. 0.57 ± 0.68, *p*<0.001), solid component volume (0.59 ± 0.83 vs. 0.08 ± 0.17, *p*<0.001), mass (1.17 ± 1.08 vs. 0.29 ± 0.35, *p*<0.001), mass of solid components (0.51 ± 0.75 vs. 0.06 ± 0.15, *p*<0.001), and percentage of solid components (14.00 ± 14.55 vs. 28.09 ± 22.55, *p*<0.001) were relatively larger in the IAC group compared to the AIS/MIA group in terms of in the 3D metrics. Among the morphological signs, lobar signs (77.1% vs. 35.1%, *p*<0.001), spiculation signs (72.6% vs. 27.4%, *p*<0.001), and pleural traction signs (24.2% vs. 1.3%, *p*<0.001) were more frequently seen in the IAC group (**Table 2**). As to the variables of the density histogram, the IAC group had greater values of variance (×10,000) (2.51 ± 1.23 vs. 1.87 ± 1.20, *p*<0.001) and entropy (8.63 ± 0.46 vs. 7.94 ± 0.55, *p*<0.001), and relatively smaller skewness (0.69 ± 0.54 vs. 0.83± 0.49, *p*<0.001) and kurtosis (3.09 ± 1.49 vs. 3.39 ± 1.50, *p*=0.003) than the AIS/MIA group.

### Independent risk factors for IAC in the training cohort

The clinical characteristics and imaging information were balanced in the training and validation cohorts (**Supplementary Table S1**). The clinical information, 3D imaging metrics, morphological signs, and density histogram-related metrics were included in the multivariate logistic regression analysis. The mass of PSN was calculated based on the mean density and volume. Moreover, there was multi-collinearity between the three-dimensional metrics of PSN as a whole and the solid component, which is generally considered to represent the invasive portion of PSN. In order to avoid the impact of multi-collinearity on the multivariate analysis, the mass-related metrics were excluded from the subsequent analysis, and the three-dimensional metrics of solid component were preferentially selected for the analysis. The results of the multivariate logistic regression analysis showed that solid mean density [odds ratio (OR), 1.015; 95% CI, 1.004–1.027, p=0.009], solid volume (OR, 1.085; 95% CI, 1.028–1.143, *p*=0.003), diameter (OR, 1.183; 95% CI, 1.085–1.291, *p*<0.001), variance (OR, 0.605; 95% CI, 0.410–0.893, *p*=0.011), and entropy (OR, 2.008; 95% CI, 2.750–7.717; p=0.039) were independent risk factors for the pathological invasiveness of PSN as IAC (**Table 3**).

**Table 3:** Univariate and multivariate analyses of the ability of each factor in predicting invasive adenocarcinoma in the training cohort.

Features	Univariate analysis (input method)		Multivariate analysis (input method)	
	OR (95% confidence interval)	*p*-value	OR (95% confidence interval)	*p*-value
Gender		0.157		
Women	Reference			
Male	1.276 (0.911-1.788)			
Age (years)	1.042 (1.025-1.058)	**<0.001**	1.009 (0.990-1.029)	0.358
Smoking history		**0.006**		0.087
Non-smokers	Reference		Reference	
Current/previous smokers	1.867 (1.194-2.920)		1.626 (0.932-2.837)	
Family history of malignant tumors		0.156		
Yes	1.453 (0.867-2.435)			
None	Reference			
Solid mean density (HU)	1.015 (1.012-1.019)	**<0.001**	1.015 (1.004-1.027)	**0.009**
Solid volume (cm^3^)	554.326 (122.713-2504.019)	**<0.001**	1.085 (1.028-1.143)	**0.003**
lobar sign		**<0.001**		0.933
None	Reference		Reference	
Yes	6.248 (4.463-8.775)		0.971 (0.487-1.934)	
Spiculation sign		**<0.001**		0.266
None	Reference		Reference	
Yes	6.958 (4.916-9.850)		1.490 (0.738-3.008)	
Pleural traction sign		**<0.001**		0.263
None	Reference		Reference	
Yes	22.585 (7.121-71.637)		2.092 (0.574-7.615)	
Diameter (mm)	1.341 (1.278-1.407)	**<0.001**	1.183 (1.085-1.291)	**<0.001**
Percentage of solid components (%)	52.754 (18.592-149.685)	**<0.001**	0.557 (0.033-9.455)	0.686
CT Histogram				
Variance (×10,000)	1.617 (1.370- 1.909)	**<0.001**	0.605 (0.410-0.893)	**0.011**
Skewness	0.624 (0.467-0.833)	**0.001**	0.447 (0.122-1.6273)	0.222
Kurtosis	0.898 (0.817-0.986)	**0.025**	1.001 (0.717-1.396)	0.997
Entropy	13.293 (8.693-20.236)	**<0.001**	2.008 (1.036-3.891)	**0.039**

Bold values denote statistical significance at the P < 0.05 level.

### Optimal model for predicting IAC in PSNs

Three prediction models (clinical, histogram, and integrated model) were built based on the results of the multivariate logistic regression analysis. The clinical model incorporated the diameter of the nodule and CT signs with clinical significance (spiculation sign and pleural traction sign), while the histogram model included AI-derived histogram features, which were independent predictors for the invasion status (variance, entropy, the mean density of the solid component, and volume of the solid component). Finally, we established an integrated model incorporating independent variables in both the clinical and histogram models.

**Figure 3:**
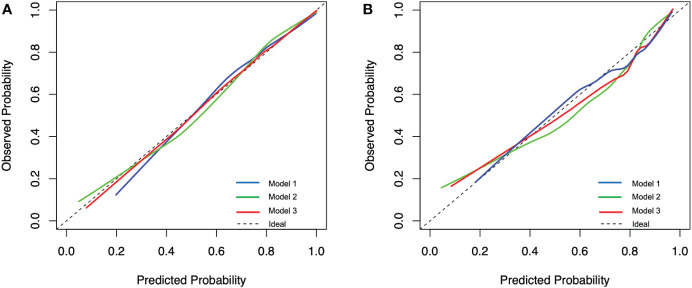
Calibration plot of the prediction models for predicting invasive adenocarcinoma lesions from the training cohort **(A)** and the validation cohort **(B)**. Model 1, clinical model; Model 2, histogram model; Model 3, integrated model.

**Figure 4:**
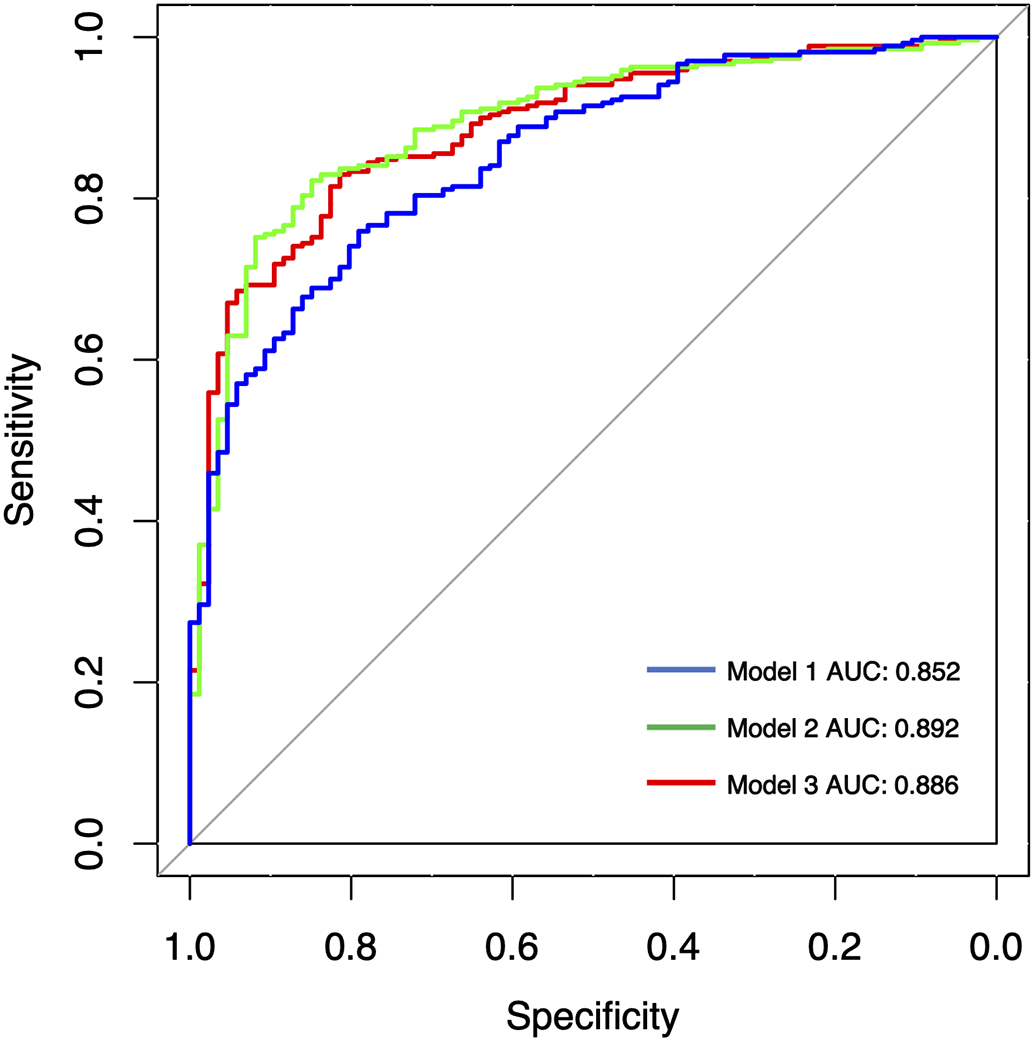
Receiver operating characteristic (ROC) analysis of assessing pathological invasiveness of PSN. AUC, area under the ROC curve; Model 1, clinical model; Model 2, histogram model; Model 3, integrated model.

The calibration curves presented good consistency in three models between the predicted and actual observed probability of IAC in the training and validation cohort (**Figure 3**). The AUCs in the training cohort and validation cohort indicated a better discriminative ability of the histogram model (AUC=0.892) compared with the clinical model (AUC=0.852) and integrated model (AUC=0.886), although the clinical model showed better AIC and BIC values (**Figure 4**; **Table 4**). Delong’s test showed that the histogram model (model 2) had a significantly higher AUC than the clinical model (*p*=0.005), while the comparison between histogram model and integrated model fell short of statistical significance (*p*<0.05, **Supplementary Table S2**).

**Table 4:** Diagnostic performance of three predictive models for predicting invasive adenocarcinoma lesions.

	AUC 1	AUC 2	AIC	BIC	Sensitivity	Specificity
Model 1 Clinical model (diameter, spiculation sign, pleural traction sign)	0.833	0.852	699.102	717.998	0.759	0.791
Model 2 Histogram model (variance, entropy, the mean density of the solid component, volume of the solid component)	0.833	0.892	694.037	717.656	0.822	0.849
Model 3 Integrated model (Clinical model+ Histogram model)	0.833	0.886	673.056	710.847	0.830	0.814

AUC 1, 10-fold AUC result based on training cohort; AUC 2, results based on validation cohort. AIC, Akaike Information Criterion; BIC, Bayesian Information Criterion.

## Discussion

In recent years, the digital processing of medical images becomes a current hot spot. The quantitative analysis of CT information has advanced the understanding of the internal structure of lung nodules. Especially the artificial intelligence technology represented by deep learning has been widely used in the field of medical imaging. The screening, analysis, and diagnosis of lung nodules based on CT images have entered the era of precision and intelligence. The clinical diagnosis process and efficiency of pulmonary nodules have been greatly improved (19, 22). At the same time, AI’s accurate lesion segmentation, lesion volume measurement, and sensitive sign recognition also provide richer features and more reproducible objective indicators for accurate prediction of the pathological invasiveness of lung nodules (23).

In view of the fact that most of the previous reports took SSN as the whole study object, we investigated the clinical and imaging indicators associated with the degree of pathological invasiveness only in PSNs, which are more in need of surgical intervention. The multivariate analysis revealed that diameter, solid mean density, solid volume, variance, and entropy were independent risk factors of IAC in the training cohort. Furthermore, we constructed three prediction models of IAC using clinical information, AI-based density histogram features, and both from a large sample cohort. The histogram model constructed by AI-based density histogram features showed the best performance in assessing the pathological invasiveness of PSNs in the validation cohort (AUC=0.892). The possible reason that the integrated model has lower AUC value than the histogram model is that the addition of morphological signs may reduce the specificity.

In the selection of the differentiation threshold between solid and ground glass components, previous studies found that the sensitivity of solid component recognition gradually decreases and the specificity gradually increases with the gradual increase in the set threshold in a certain range, while the recognition effect of the ground glass component is the opposite. One of the thresholds with a better integrative performance of solid component recognition is −300 HU (18, 24). Therefore, −300 HU was used as the threshold for the classification of ground glass components and solid components in this study.

Considering that the solid component of PSN tends to correspond to the fraction of histological invasive growth, we preferentially selected the solid component indicators in the multivariate logistic regression analysis. Yu et al. from Korea suggested that the assessment effect of high-dimensional indicators was better than that low-dimensional indicators (3D > 2D > 1D) in the assessment of pathological invasiveness in the PSN (25). Many previous studies have demonstrated the correlation between the diameter of the solid component or CTR and the pathological invasiveness of PSNs (26, 27). However, there are still relatively few studies on the correlation between three-dimensional metrics of PSN and the degree of pathological invasiveness. One study reported that mass and volume were independent factors for IAC in PSNs, but this study did not evaluate metrics such as mean density of solid components and mass of solid components (28). In the present study, the volume, mean density, and mass of both the total nodule and solid component were investigated. The volume and mean density of the solid component were independent risk factors of IAC after ruling out of the mass index to avoid the impact of multi-collinearity on the multivariate analysis. Because of the comprehensive nature of 3D metrics, especially 3D metrics of solid component, they can reflect the overall nature of nodules more comprehensively and are good indicators for assessing pathological invasiveness of PSNs.

The CT histogram metrics have undergone a gradual transition from the number and size of peaks to the integrated calculation metrics. We calculated the four most frequently used composite metrics, variance, skewness, kurtosis, and entropy to describe the distribution of the overall CT values of PSNs. The results of our study showed that although all four metrics were associated with the degree of pathological invasiveness in PSN, only variance (OR, 0.605; 95% CI, 0.410–0.893, *p*=0.011) and entropy (OR, 2.008; 95% CI, 2.750–7.717; *p*=0.039) were independent risk factors for IAC. Most of the current studies on CT histogram indicators and the degree of pathological invasiveness of SSN did not distinguish pGGN and PSN, with SSN as a whole study. Yagi et al. also showed that AIS/MIA had significantly higher skewness and kurtosis and relatively lower values of variance and entropy compared to IAC, and the results of multivariate logistic regression analysis showed that entropy was an independent risk factor between the two groups (29). However, when Chae et al. explored imaging metrics for assessing invasiveness in PSNs, they found that a higher kurtosis was an independent factor in distinguishing pre-invasive lesions (AAH/AIS) from invasive lesions (MIA/IAC) (9). It follows that less invasive lesions tend to show lower variance and entropy and greater kurtosis and skewness in the univariate analysis, representing lower heterogeneity and a more concentrated state at lower CT values. Entropy and variance are important indicators for differentiating pathological invasiveness in PSNs, but the grouping of pathological types expected to be differentiated should also be considered.

Beyond the first-order histogram features, the second- and higher-order texture features extracted by radiomics techniques were also integrated in the assessment of invasiveness among SSNs (30–32). Li et al. obtained the CT texture features of 109 SSNs and found that the surface area feature and the extruded surface area feature could be predictors of IACs compared with MIAs (33). Wu et al. analyzed the association between CT−based conventional features/selected radiomic features and histological invasiveness of 203 SSNs in the training cohort and 57 SSNs in the validation cohort. The diagnostic performance of the radiomic feature was as great as that of quantitative CT feature (nodular size and solid component) (34). Recently, they further studied the radiomics features of 260 SSNs and constructed a LASSO-derived model integrating semantic–radiomic features, which showed excellent diagnosis performance (AUC, 0.957) to predict invasive SSNs (35).

In the majority of above studies, the SSNs were investigated as a whole, while only three studies focused on the assessment of invasiveness in PSNs by radiomics technology. Chae et al. constructed a three-layered artificial neural networks model with mean attenuation, standard deviation of attenuation, mass, kurtosis, and entropy in 86 PSNs (9). Even though the model showed excellent performance in differentiation of preinvasive lesions from IACs (AUC, 0.981), the small sample and lack of validation limited the model’s application. Weng et al. identified that four radiomics features, including MaxIntensity, RMS, ZonePercentage, and Long-RunEmphasis_angle0_offset7, were the best discriminators to predict invasiveness of PSNs (12). A nomogram that integrated lesion shape and radiomic signature could achieve a satisfactory AUC of 0.888 (12). In a retrospective multicenter study that included 297 PSNs, Wu et al. extracted radiomic features from the different regions [gross tumor volume (GTV), solid, ground-glass, and perinodular] (13). The radiomics model based on ground-glass and solid features yielded an AUC of 0.98 on the test data set, which was significantly higher compared with the Brock, clinical–semantic features, and volumetric models. However, the model required ground-glass and solid CT radiomic features, which was too complicated to apply in clinical practice.

Nevertheless, the above studies mostly used the manual delineation method for ROI delineation and segmentation, which was time consuming and prone to inter-observer inconsistency in a real-world setting (8). Deep-learning AI techniques, as a high-efficiency and promising automatic method, has been increasingly applied in automatic lesion delineation to accelerate radiomics pipeline (32, 36, 37). In the comparison of the clinical model, histogram model, and integrated model, the histogram model constructed by AI-based histogram features demonstrated the best performance in the validation cohort (AUC=0.892), while the integrated model did not achieve better diagnostic efficiency compared with the histogram model (*p*=0.34, Delong’s test). As the histogram features could be calculated automatically by the deep-learning AI software (InferRead CT Lung), surgeons could apply the histogram model for surgical planning of PSNs in the real-world clinical practice after embedding this software in the medical imaging system.

This study has some limitations. First, this study is a retrospective single-center study with selective bias and no external validation. Second, the solid component diameter and CTR were not included in the analysis considering the measurement of solid component diameter is inherently subjective. Third, only the first order radiomic features, no more in-depth second or third radiomic features, were studied this time. There is still much room for improvement in the diagnostic performance of our model. However, the more complex radiomic features make it more difficult in practical clinical application.

## Conclusion

This large sample study demonstrated that AI-based CT histogram features could assess the pathological invasiveness of PSNs accurately. With further external validation in the future, this convenient histogram model is very promising to be applied to guide clinical treatment decisions of PSNs in the real-world setting.

## Data availability statement

The raw data supporting the conclusions of this article will be made available by the authors, without undue reservation.

## Ethics statement

The studies involving human participants were reviewed and approved by the Peking University People’s Hospital ethics committee (2022PHB011-002). Written informed consent for participation was not required for this study by national legislation and institutional requirements.

## Author contributions

JG, QQ, HL conceived and designed the study. ZW, ZS, SC, JY, YZ and NH performed the experiments. ZW, ZS, DW and HW analyzed the data. FY, XL, YL provided critical inputs on design, analysis, and interpretation of the study. All the authors had access to the data. All authors read and approved the final manuscript as submitted.
