# Supplementary material for: Artificial-intelligence-based computed tomography histogram analysis predicting tumor invasiveness of lung adenocarcinomas manifesting as radiological part-solid nodules
Source: Front Oncol. 2023 Feb 23;13:1096453. doi: 10.3389/fonc.2023.1096453 (PMC9996279; doi:10.3389/fonc.2023.1096453)
Supplement: Supplementary file 1 [file Table_1.docx]

| Suppl TABLE 1 Clinical characteristics and imaging information of the training and validation cohorts in 1188 subjects | | | |
| --- | --- | --- | --- |
| Features | Training cohort (n=832) | Validation cohort (n=356) | P-value |
| Gender |  |  | 0.972 |
| Women | 553 (66.47) | 237 (66.57) |  |
| Male | 279 (33.53) | 119 (33.43) |  |
| Age (years) | 59.21±10.35 | 59.06±9.9 | 0.967 |
| Smoking history |  |  | 0.422 |
| Non-smokers | 673 (80.89) | 295 (82.87) |  |
| Current/previous smokers | 159 (19.11) | 61 (17.13) |  |
| Family history of malignant tumors |  |  | 0.708 |
| Yes | 101 (12.14) | 46 (12.92) |  |
| None | 731 (87.86) | 310 (87.08) |  |
| Solid mean density (HU) | -178.52±59.9 | -181.54±53.7 | 0.778 |
| Solid volume (cm^3^) | 0.48±0.80 | 0.42±0.66 | 0.767 |
| lobar sign |  |  | 0.088 |
| Yes | 566 (68.03) | 224 (62.92) |  |
| None | 266 (31.97) | 132 (37.08) |  |
| Spiculation sign |  |  | 0.616 |
| Yes | 513 (61.66) | 214 (60.11) |  |
| None | 319 (38.34) | 142 (39.89) |  |
| Pleural traction sign |  |  | 0.919 |
| Yes | 154 (18.51) | 65 (18.26) |  |
| None | 678 (81.49) | 291 (81.74) |  |
| Diameter (mm) | 16.60± 5.91 | 16.48± 5.98 | 0.798 |
| Percentage of solid components (%) | 0.25±0.22 | 0.24±0.21 | 0.736 |
| CT Histogram |  |  |  |
| Variance (×10000) | 2.36±1.30 | 2.32±1.13 | 0.764 |
| Skewness | 0.72±0.54 | 0.74±0.51 | 0.348 |
| Kurtosis | 3.17±1.56 | 3.16±1.36 | 0.668 |
| Entropy | 8.47±0.56 | 8.45±0.59 | 0.983 |
| Pathology |  |  | 0.599 |
| AIS/MIA | 213 (25.6) | 86 (24.16) |  |
| IAC | 619 (74.4) | 270 (75.84) |  |

Data are expressed as mean ± standard deviation or as a number (percentage). AIS, adenocarcinoma in situ; MIA, minimally invasive adenocarcinoma; IAC, invasive adenocarcinoma.

| Suppl TABLE 2 Delong's test based on the AUC of validation cohort | | |
| --- | --- | --- |
|  | Delong test |  |
| Model 2 vs. Model 1 | P=0.0050 |  |
| Model 2 vs. Model 3 | P=0.3408 |  |
| Model 3 vs. Model 1 | P=0.0002 |  |

AUC, area under the receiver operating characteristic curve. Model 1, Clinical model; Model 2, Histogram model; Model 3, Integrated model.
